# Comparative Efficacy of Magnesium and Potassium Towards Cholesterol and Quality of Life in Patients With Type 2 Diabetes Mellitus: A Randomised Single‐Blinded Controlled Clinical Trial

**DOI:** 10.1002/edm2.511

**Published:** 2024-10-15

**Authors:** Sidra Khalid, Riffat Mehboob, Syeda Shazia Bokhari, Muhammad Ali, Ambreen Shabbir, Khurram Mehboob, Hafsa Adnan, Mohammed Matoog Karami, Hani Shalabi, Bander Alshehri

**Affiliations:** ^1^ Institute of Diet & Nutritional Sciences The University of Lahore Lahore Pakistan; ^2^ Lahore Medical Research Center Lahore Pakistan; ^3^ National Heart Lung and Blood Institute National Institutes of Health Bethesda MD USA; ^4^ Department of Biology Lahore Garrison University Lahore Pakistan; ^5^ Department of Clinical Physiology, Faculty of Medicine King Abdulaziz University Jeddah Saudi Arabia; ^6^ Department of Internal Medicine, Faculty of Medicine University of Jeddah Jeddah Saudi Arabia

**Keywords:** cholesterol, magnesium, potassium, quality of life, type 2 diabetes mellitus

## Abstract

Previous studies exploring the relationship between dietary potassium and magnesium intake and the risk of type 2 diabetes mellitus (T2DM) have yielded inconsistent results.

**Objective:**

The present study was designed to compare the effect of magnesium, potassium and both (potassium and magnesium combined) on cholesterol levels and quality of life (QoL) among patients with T2DM.

**Methods:**

A randomised controlled trial (single blinded) was conducted at The University of Lahore and Lahore Medical Research Center (LMRC). The sample size was 290 patients with T2DM, who were divided into four groups: Group I (T1) that received control/placebo; Group II (T2) and Group III (T3) received magnesium and potassium supplements, respectively; and Group IV (T4) received both magnesium and potassium supplements. Blood samples were taken from all patients before and after 60 days of supplementation to determine the levels of K^+^, Mg^2+^ and cholesterol using a chemistry analyzer (photometer 5010 v5+).

**Results:**

There was a decrease in mean cholesterol levels in all groups after the treatment, with the largest reduction (224.9 ± 61.92 to 163.4 ± 48.38) seen in the T3 group, that received potassium supplements. A significant increase in the social QoL, indicated by a *p* value change from 0.06 before medical intervention to 0.000 after medical intervention, was observed. *p* value was significant (<0.05) between pre‐ and post‐QoL within the T2 (Mg) and T3 (K) treatment groups.

**Conclusions:**

The overall decrease in cholesterol levels and improvement in the social QoL after treatment imply that magnesium‐ and potassium‐based formulations prove beneficial in combating hyperlipidaemia in patients with T2DM.

**Trial Registration:** NCT04642313

## Graphical abstract

Supplemental magnesium and potassium significantly reduced cholesterol levels and improved social quality of life in individuals with type 2 diabetes mellitus, suggesting potential pathways for controlling hyperlipidaemia and enhancing overall health outcomes.

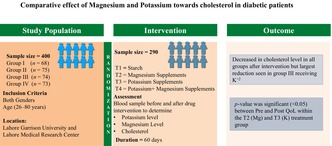

## 1 Introduction

Diabetes is considered as the most concerned public health issue. According to an article by ‘The News’, Pakistan ranks third in the world in diabetes prevalence after China and India [1]. The International Diabetes Federation reported that, in 2021, 26.7% of adults in Pakistan are affected by diabetes [2]. It is a condition related to the metabolism of carbohydrates in which there is a persistently elevated blood glucose level because of inadequate insulin secretion or activity. The two types of diabetes are type 1 diabetes mellitus (T1DM) and type 2 diabetes mellitus (T2DM). T1DM commonly manifests in childhood and is mediated by immunological systems [3]. T2DM is one of the most common metabolic disorders worldwide and its development is primarily caused by a combination of two main factors: defective insulin secretion by pancreatic β‐cells and the inability of insulin‐sensitive tissues to respond to insulin [4]. Diabetes raises the possibility of complications from a number of different diseases. Tooth decay, kidney and eye diseases, neuropathy, cardiovascular and blood vessel diseases, and nerve damage are all brought on by persistently elevated blood sugar levels [1].

Magnesium is vital to the body's molecular, biochemical, physiological and pharmacological processes [5]. Numerous metabolic investigations have shown that magnesium supplementation has an advantageous impact on how insulin works and in the metabolism of glucose, the pathway involving hypomagnesaemia and diabetes mellitus is yet unknown [6]. According to research, deficiency of magnesium has been related to a range of chronic diseases, including type 2 diabetes, Alzheimer disease, migraine headaches and stroke [7, 8]. Hypomagnesaemia is recognised to occur more frequently in patients with type 2 diabetes. Magnesium shortage has been demonstrated to impair glycaemic control through altered cellular glucose transport, decreased pancreatic insulin production and impaired post‐receptor insulin signalling. There is also substantial evidence associating hypomagnesaemia to a variety of diabetic problem [9]. Magnesium insufficiency has been linked to atherosclerotic disease in epidemiological and experimental research. It can also produce coronary artery hyperreactivity to vasoconstrictive stimulation. Magnesium levels should be checked in patients with vascular disease [10].

Potassium is an essential mineral, which plays major roles for the resting membrane potential and intracellular osmolarity [11]. According to research, hypokalaemia, or decreased serum potassium brought on by diuretics, is linked to impaired glucose tolerance and a higher risk of developing diabetes in hypertensive people [12, 13]. Significant decreases in serum potassium and total body potassium in a small group of healthy people experimentally fed a low‐potassium diet have been hypothesised to cause glucose intolerance and impair insulin secretion, both of which are regulated by ATP‐sensitive potassium channels [14, 15]. It was discovered that White Americans and African Americans have an inverse relationship between serum potassium levels and an increased risk of T2DM that is unrelated to the use of diuretics [16]. Numerous epidemiological studies have found that potassium consumption and blood pressure are inversely associated, with blood pressure falling as potassium intake rises. A significant independent factor affecting the population's blood pressure was potassium consumption, as determined by 24‐h urinary potassium excretion, according to the big multinational study on electrolytes and blood pressure [17].

It is important to identify modifiable risk factors of T2DM to prevent this public health problem. Prospective observational studies have shown the importance of diet and dietary patterns in the prevention and management of T2DM [18]. Micronutrients are usually being ignored in the diet due to inappropriate dietary patterns or lack of awareness. This study hypothesised that magnesium and potassium supplementation affect the cholesterol and quality of life (QoL) in patients with T2DM. This study will help determine the combined effect of magnesium and potassium towards cholesterol and QoL in T2DM.

## 2 Methods

### 2.1 Study Design and Duration

A single‐blind, randomised controlled trial was conducted for a duration of 9 months from September 2022 to May 2023.

### 2.2 Ethical Approval

Ethical approval (IRB‐UOL‐FAHS/760/2020) was gained from the Institutional review board (IRB), Faculty of Allied Health Sciences, The University of Lahore.

### 2.3 Sample Size

The sample size was 290, which was estimated using the prevalence of type 2 diabetes that was 16.98% at 8% margin of error and 95% confidence level.

### 2.4 Inclusion and Exclusion Criteria

All patients with diabetes with both genders and age between 26 and 80 years were included in the study. Individuals with the following conditions were not included: pregnancy, nephropathy, alcohol consumption, history of chronic liver disease and psychiatric disorders.

### 2.5 Informed Consent

A sterilised syringe was used to collect a blood sample before and after supplement treatment at Alakhuwat Foundation. The blood was collected using a venipuncture technique, and the participant provided their informed written consent.

### 2.6 Outcome Measures

Body mass index (BMI) was calculated using the standard formula (weight in kilograms/height in square metre: kg/m^2^) to exclude obesity.

### 2.7 Study Groups and Supplementation

To test the effects of potassium and magnesium supplements on cholesterol in patients with T2DM, four groups were formed that were exposed to medical treatment for 60 days. Group I (T1) was the control/placebo group that consisted of patients who received starch (Calip D 250 mg × 2) supplements; Group II (T2) received magnesium supplements (Ostin 250 mg × 2); Group III (T3) received potassium supplements (Paravit 250 mg × 2); Group IV (T4) received both Mg and K medication in form of Bionta (250 mg × 2). Different tests were performed in Lahore Medical Research Lab before and after medication, and the levels of K^+^, Mg^2+^ and cholesterol were evaluated. Test was performed using a chemistry analyzer (photometer 5010 v5+). To assess QoL, the WHOQOL‐BREF questionnaire, which consists of 20 items, was adapted [19]. Throughout the supplementation period, participants were monitored continuously for unusual symptoms or health issues.

### 2.8 Statistical Analysis

Descriptive statistics, multiple *t*‐tests and one‐way ANOVA were performed using Prism GraphPad (version 8.02).

### 2.9 Consort Flow Diagram

**FIGURE 1:**
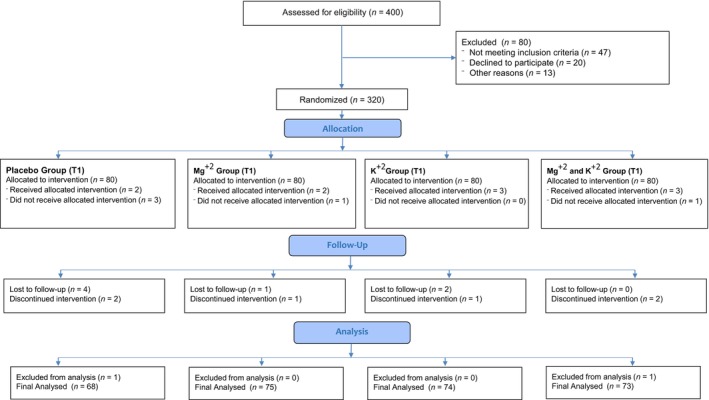
Consort flow diagram showing the details of randomized controlled trial (RCT).

A consort flow diagram is provided that is defining the steps of randomised controlled trial (RCT) and how the participants were shortlisted for final trail (Figure 1).

## 3 Results

The minimum age was 25 years and the maximum age was 85 years. The mean age of the participants was 51.43 ± 11.33. There were 195 (67.5%) females and 95 (32.7%) males among the 290 participants (Table 1).

**TABLE 1:** Demographics of age and gender.

Variables	Frequency (%)
Gender	
Male	95 (32.7%)
Female	195 (67.5%)
Mean age (Mean ± SD)	51.43 ± 11.33

Table 2 depicts the change in the cholesterol levels before and after medical interventions among the groups. The placebo group (T1) shows significant difference (*p* = 0.003 < 0.05) in cholesterol levels before (I) and after (II) dose administrations. However, this difference is less significant as compared to the magnesium (T2) group (*p* = 0.00006), potassium group (*p* < 0.000001) and the magnesium + potassium group (*p* = 0.002), with phase II mean cholesterol levels of 171 ± 52.69, 163.4 ± 48.43 and 196.2 ± 54.06. This sets the seal that magnesium and potassium intake play efficient roles in managing high cholesterol levels of patients with diabetes with a major effect created by potassium T3 medication. Overall, the Mg and K medication intercession of the T1–T4 groups in managing cholesterol levels that are seen escalated in T2DM gives statistically significant results (*p* = 0.000001).

**TABLE 2:** Medication group‐based differences in cholesterol levels.

Groups	*N* (%)	Cholesterol I	Cholesterol II	*p* value
T1	68 (23.45)	217.6 ± 68	183.2 ± 68.55	0.003
T2	75 (25.8%)	211 ± 65.41	171 ± 52.69	0.00006
T3	74 (25.5%)	224.9 ± 61.92	163.4 ± 48.38	<0.000001
T4	73 (25.1%)	222.5 ± 49.06	196.2 ± 54.06	0.002495
*p* value		**0.5277**	**0.0028**	

*Note:* Bold value is describing the difference among treatment groups.

**FIGURE 2:**
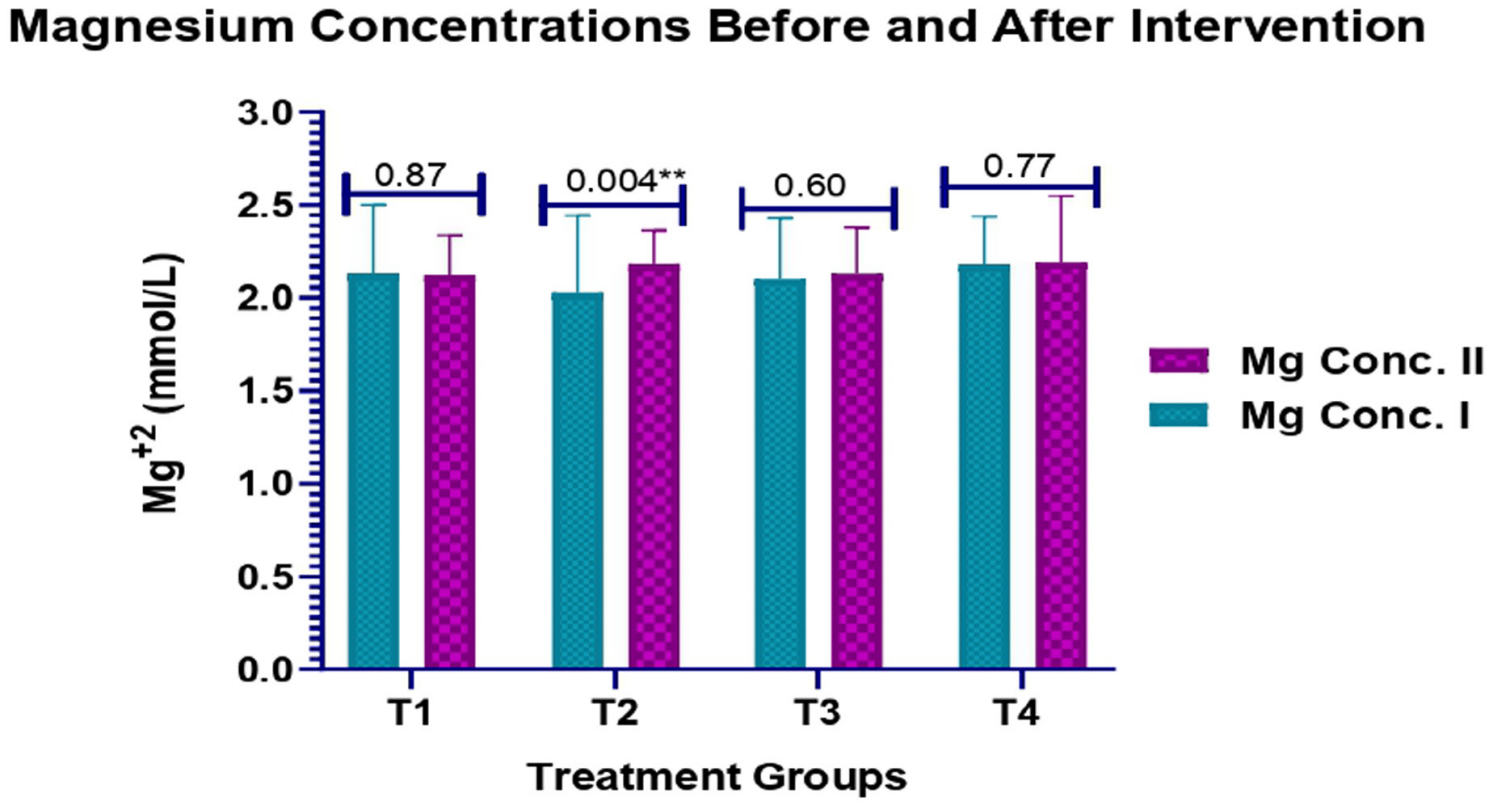
Changes in magnesium concentrations before and after medical interventions.

The differences in the levels of Mg in patients with T2DM before and after interventions within each medication group were analysed. As it is the measurement of the magnesium levels, the difference is the most significant in the T2 group showing before and after intervention mean values of 2.027 ± 0.4182 and 2.181 ± 0.182, respectively (*p* = 0.004). The T1, T3 and T4 groups resulted in statistically non‐significant values of pre‐ and post‐Mg levels. The statistical differences in pre‐ and post‐Mg levels with respect to the medication groups are also insignificant with *p* values of 0.12 and 0.072, respectively, that is less than the cut‐off value of 0.05. Inferentially, the medication strategy has no effects on the magnesium levels as indicated by insignificant *p* value = 0.071 (Figure 2).

**FIGURE 3:**
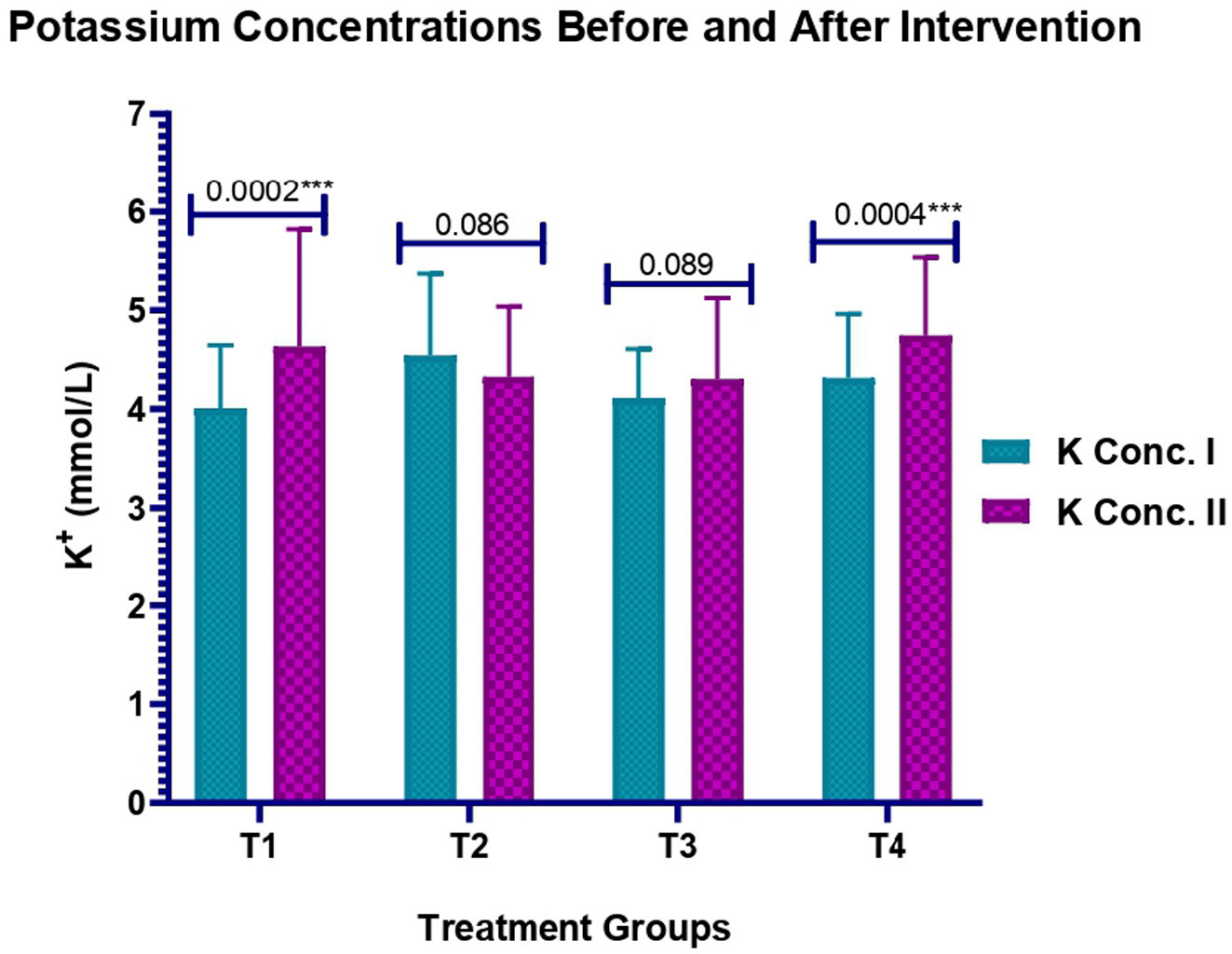
Changes in potassium concentrations before and after medication interventions.

The non‐conformities in the levels of K in patients with T2DM before and after interventions of medication in each group were measured. Only the combined dose of magnesium and potassium in the T4 group resulted in the emergence of statistically significant difference (*p* = 0.00045) having an average quantity measure of 4.316  ±  0.6479 before and 4.745  ±  0.7901 after which specifies the effectiveness of K and Mg combined influence on K levels of patients with T2DM. The overall significant *p* value of 0.00047 which highlights that the intervention has the potential positive consequences on the K levels of patients with diabetes. The statistical difference within the pre–K levels is significant as denoted by *p* = 0.00002 < 0.05 and that within mean post–K levels is slightly close to significance as represented by *p* = 0.06 >0.05 (Figure 3).

**FIGURE 4:**
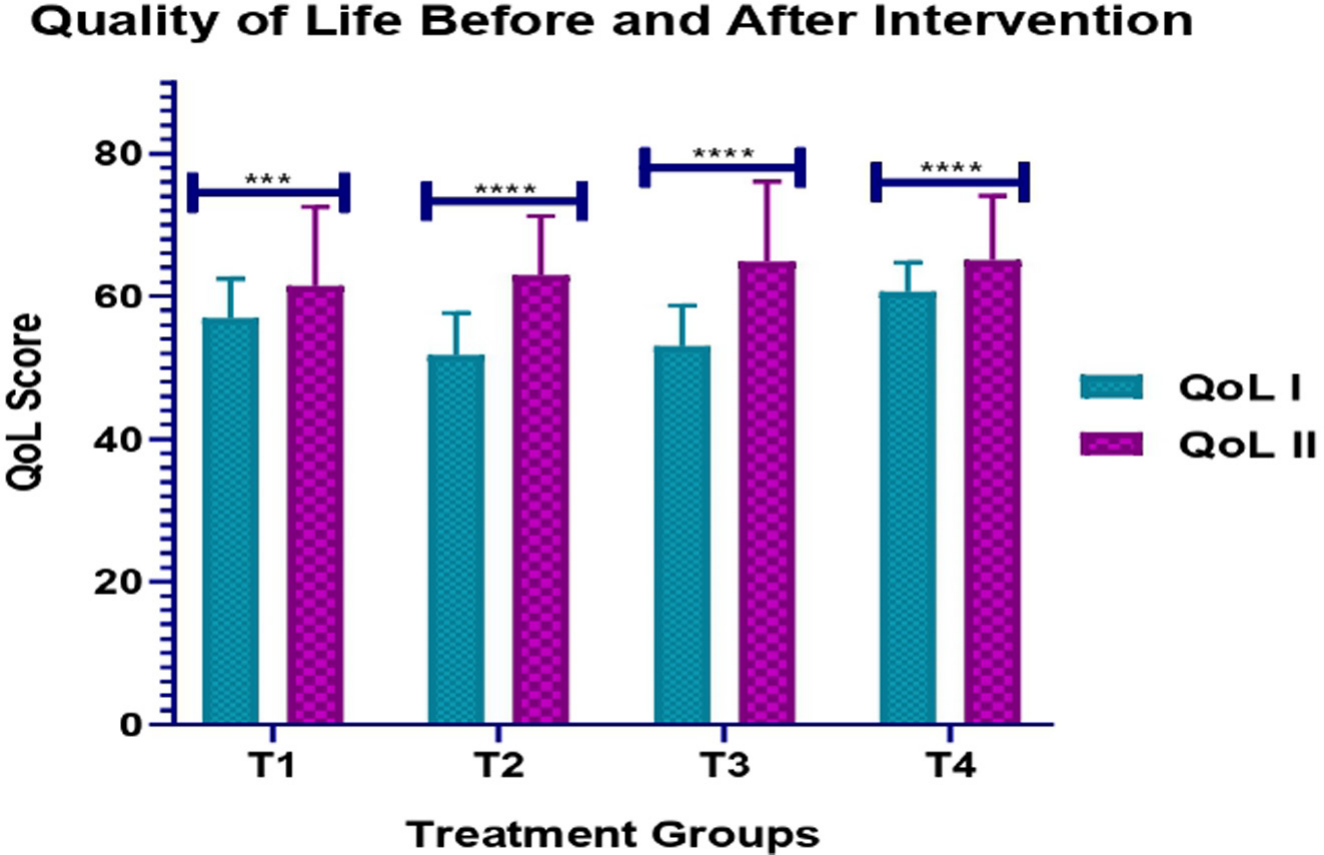
QoL of patients in different medication groups before and after treatment.

QoL of patients within each medication group before and after dose inculcation was scrutinised. Upon analysis, patients in the placebo group (T1) who were administered with starch doses displayed a significant difference in means of pre‐ and post‐QoL scores (*p* = 0.003 < 0.05). The effectiveness of proposed medication strategy on the QoL of subjects intervened with magnesium, potassium and combined supplementation is evident by the more significant *p* values that are <0.000001, <0.000001 and 0.00029, respectively, with an equal and greatest effectiveness observed as a result of Mg and K supplementation. Apart from this, the T2–T4 groups dispensed with Mg, K and Mg + K medicines, respectively, resulted in an increase in the QoL scores with the highest elevation (51.81 ± 5.865) seen in the Mg group (Figure 4).No side effects were observed in any of the participants in all groups throughout the study period.

## 4 Discussion

The present study includes the descriptive statistics for various variables including age, gender and biomarkers such as random blood sugar levels, magnesium levels, potassium levels (K^+^) and cholesterol levels before and after treatment. All four groups experienced a decrease in the BS/R variable after the treatment, as the post‐treatment values are lower than the pre‐treatment values for all groups. However, the magnitude of the decrease varied across the different treatment groups. The T3 group, that received magnesium supplements, had the highest pre‐treatment mean cholesterol value followed by a highest decrease post‐treatment. The least decrease was observed in the T4 group (26 units in mean value) that were administered with combined medication of Mg and K. Li J et al. reported that there is frequent deficiency of magnesium and potassium in patients with diabetes; therefore, dietary potassium and magnesium intake are important to mitigate risk factors for T2DM [20]. In our study, the levels of Mg^2+^ did not significantly change in the T1 group, T3 group and T4 group from pre‐ to post‐treatment, while there was an increase in Mg^2+^ levels in the T2 group. Specifically, the T2 group had the largest increase in Mg^2+^ levels pre‐ and post‐treatment of 0.154 units in mean values. Similar results were seen in another study by Afridi et al., who found that Mg^2+^ concentrations of 65.3 ± 102 and 67.5 ± 98.2 mg/L in female and male patients with diabetes were significantly higher than normal values [21]. The levels of K^+^ increased in the T1, T3 and T4 groups but decreased in the T2 group after the treatment. However, there are some differences between the groups. The T2 group had the highest pre‐treatment mean K^+^ value and the T1 group had the lowest pre‐treatment mean K^+^ value and the largest increase in K^+^ after the treatment. The T3 and T4 groups had increased pre‐ and post‐treatment mean K^+^ values. Afridi et al. also studied potassium levels in patients with diabetes and found that the levels of potassium were significantly higher (*p* = 0.05) than the normal levels in patients with diabetes of both genders.

The treatment had a major impact on cholesterol levels in all the four groups. Specifically, there was a decrease in mean cholesterol levels in all the groups after the treatment, with the largest reduction seen in T4 group. Similar results were found by Eriksson J et al., who studied the effect of ascorbic acid and magnesium supplements in 56 patients with diabetes [22]. They observed beneficial effects of above‐mentioned supplements on triglycerides and cholesterol levels (*p* < 0.05) in patients with diabetes. The research concluded a significant increase in the social QoL of patients with diabetes. The *p* value was 0.000, which is more significant. Our results confirm past research showing the efficacy of magnesium in the management of stress, anxiety and depression [23, 24, 25, 26, 27, 28, 29].

By managing nutrients, which includes probiotics, prebiotics, vitamins and micronutrients, will allow to create more all‐encompassing strategies to reduce the difficulties associated with diabetes. Since the rise of civilised societies, food processing and cooking have resulted in a significant decrease in the potassium content of food, along with a significant increase in the consumption of processed foods; in contrast, consumption of fruits and vegetables has decreased, leading to a significant reduction in potassium consumption and a significant increase in salt intake [30]. In a similar way, dark, leafy green vegetables and whole grains, including white potatoes, are rich in magnesium [31]. Patients with diabetes can show notable gains in potassium and magnesium levels if they consume more fruits and vegetables. According to Ghoreishi et al., consumption of ginger for 3 months improved serum insulin levels, HDL cholesterol levels and insulin resistance [32]. Similarly, the association was found between particular vitamins and diabetes, pointing out that because of problems with glucose metabolism and oxidative stress, people with diabetes frequently have reduced levels of antioxidant vitamins (A, C and E) [33]. A same kind of research was conducted where the effect of Mg^+2^, K^+2^ and their combine effect was analysed on cholesterol, liver and kidney markers. After the intervention, the levels of cholesterol were noticeably reduced and settled within the normal range. This improvement was observed in all liver and kidney markers. There was no significant difference in ALT, AST, urea and creatinine levels before and after the intervention [34]. It is clear that altering food habits and consuming more probiotics and prebiotics may prove to be beneficial supplementary measures for the management of type 2 diabetes [35].

In conclusion, this investigation looked at the impact of magnesium and potassium supplements on cholesterol levels and QoL in patients with type 2 diabetes. The treatment had a significant effect on cholesterol levels, with the largest reduction seen in the group that received potassium supplement. The study showed a significant increase in the social QoL after medication followed by the effect on the psychological domain of QoL that needs explanation beyond the pre‐set symptoms. These findings suggest that magnesium and potassium supplements may be beneficial in improving the lipid profile and overall QoL of patients with T2DM. Further studies are required with larger sample sizes to enhance the validity of findings. Since the duration of intervention was 60 days in this study, a longer follow‐up is required to assess long‐term effectiveness of potassium and magnesium.

## Author Contributions

**Sidra Khalid:** conceptualization, sampling, methodology, write‐up, analysis. **Riffat Mehboob:** conceptualization, project administration. **Syeda Shazia Bokhari:** methodology, writeup. **Muhammad Ali:** write‐up and analysis. **Ambreen Shabbir:** write‐up, review, editing. **Khurram Mehboob:** analysis, sampling, record keeping and follow‐up. **Hafsa Adnan:** write‐up, sampling. **Mohammed Matoog Karami:** analysis, review and editing. **Hani Shalabi:** interpretation, analysis, review. **Bander Alshehri:** critical review, analysis.

## Ethics Statement

Ethical approval (IRB‐UOL‐FAHS/760/2020) was gained from the Institutional review board (IRB), Faculty of Allied Health Sciences, The University of Lahore.

## Consent

All participants voluntarily agreed to take part in the study, and informed written consent was obtained from each participant. All measures were taken to ensure participant confidentiality and informed consent.

## Conflicts of Interest

The authors declare no conflicts of interest.

## Data Availability

All the data of study will be provided upon request.
